# Exploring the potential of open big data from ticketing websites to characterize travel patterns within the Chinese high-speed rail system

**DOI:** 10.1371/journal.pone.0178023

**Published:** 2017-06-02

**Authors:** Sheng Wei, Jinfu Yuan, Yanning Qiu, Xiali Luan, Shanrui Han, Wen Zhou, Chi Xu

**Affiliations:** 1 Jiangsu Institute of Urban Planning and Design, Nanjing, China; 2 School of Architecture and Urban Planning, Nanjing, China; 3 School of Life Sciences, Nanjing University, Nanjing, China; Beihang University, CHINA

## Abstract

Big data have contributed to deepen our understanding in regards to many human systems, particularly human mobility patterns and the structure and functioning of transportation systems. Resonating the recent call for ‘open big data,’ big data from various sources on a range of scales have become increasingly accessible to the public. However, open big data relevant to travelers within public transit tools remain scarce, hindering any further in-depth study on human mobility patterns. Here, we explore ticketing-website derived data that are publically available but have been largely neglected. We demonstrate the power, potential and limitations of this open big data, using the Chinese high-speed rail (HSR) system as an example. Using an application programming interface, we automatically collected the data on the remaining tickets (RTD) for scheduled trains at the last second before departure in order to retrieve information on unused transit capacity, occupancy rate of trains, and passenger flux at stations. We show that this information is highly useful in characterizing the spatiotemporal patterns of traveling behaviors on the Chinese HSR, such as weekend traveling behavior, imbalanced commuting behavior, and station functionality. Our work facilitates the understanding of human traveling patterns along the Chinese HSR, and the functionality of the largest HSR system in the world. We expect our work to attract attention regarding this unique open big data source for the study of analogous transportation systems.

## Introduction

Every second, a massive amount of data is recorded using information and communication tools, such as GPS, mobile phones, Wi-Fi, and social media. These so-called ‘big data’ contain valuable information regarding how people interact between each other and with their environment [1], providing exciting opportunities for a detailed look into the structure, function and dynamics of human systems, and to understand spatiotemporal patterns of human behavior at both the macro and micro level [2]. In the meantime, development of Internet technology, high-performance computing and data storage capacity now allow us to harness big data efficiently and effectively. These technological advancements have largely boosted the collection and application of big data, profoundly impacting a wide range of fields.

Big data on human mobility have proven especially useful for characterizing fine-scale human behaviors, having led to the discovery of many interesting patterns that cannot be achieved through traditional spatially/temporally coarse surveys or official statistics. A variety of data sources, such as social media (e.g., *Twitter*), website reporting and mobile phones, have been explored to unravel, for example, social network structures, society’s mood and human traveling patterns [3–6]. Many of these data can effectively capture movement trajectories of individuals, revealing regularities in human mobility [7, 8] and providing detail in the understanding of the structure and functionality of urban systems [9]. These applications of big data have resulted in important repercussions in areas such as urban planning, the spread of disease and public health assessments [10–12]. Despite the fact that explorations of big data are increasingly diversified and intensified, so far only a small fraction of big data regarding human mobility has been investigated, compared with the massive amount of data that have already been and are currently being generated. Due to public security concerns, and privacy protection for individuals as well as corporations, most big data are exclusive to data producers or government authorities, precluding more in-depth studies. Big data that are openly accessible and effective for the public (so-called ‘open big data’) are increasingly necessary [13, 14]. It has well demonstrated that multi-sourced open big data can be used as a powerful tool for visualizing and understanding anthropogenic patterns and social dynamics at a range of spatial scales, calling for further contributions from both researchers and government authorities to the development of open big data [15].

Transportation tools are almost indispensable for human traveling nowadays. Many ‘smart’ transportation tools can now record traveling information, thus becoming an important source of human mobility data. In particular, the rapid development of public transportation systems makes it possible to retrieve traveling trajectories at an individual level. In many countries across the world, automatic fare collection systems using smart cards have been established for a wide range of public transit systems, such as railways, subways, buses, taxis and public bicycles. These systems record information showing where and when the smart-card owners tap in and out [2]. Recent studies using real-time smart cards for city subways exemplify the power of these big data [16–19]. By analyzing the data gathered from the ‘Oyster’ cards for the London Underground, Reades et al. [17] characterized a range of general properties of station flow volume to assess subway functionality and to infer traveling patterns in a large urban setting. Such analyses have been extended to multiple cities where similar smart-card systems can generate comparable datasets, resulting in the discovery of regularities and variabilities of statistical patterns of human mobility [19]. Furthermore, many exciting possibilities can arise if these big data are combined with advanced data mining techniques. For example, Ma et al. [20] developed a Density-Based Scanning Algorithm with Noise (DBSCAN) to detect transit riders’ historical travel patterns; on this basis, different groups of transit riders with varying traveling patterns were revealed using the K-Means++ clustering algorithm and the rough-set method. Such data mining approaches can also be used to assess transit network performance [21] and to characterize spatiotemporal commuting patterns of public transit riders [22]. Moreover, these smart-card data can play an important role in understanding transit ridership with the support of auxiliary environmental/anthropogenic data. For example, Li et al. [23] have constructed a decision-tree based model to quantify the relationships between the boarding/alighting demand and surrounding land use patterns at rail transit stations, and then used these yielded relationships to forecast rail transit ridership in the context of land use development. Despite the proven and the potential value, public transit data, such as the smart card data, are usually not publically available. The paucity of open big data relevant to travelers within public transit tools hinders any further in-depth study on human mobility patterns. With regard to the current social, cultural, and institutional norms against data sharing [15], it is highly necessary to explore alternative data sources that can be accessed to the public.

To address this issue, in this work we explore a useful source of public transit data that have not been investigated before: ticketing websites. With the development of the Internet, online ticketing systems have become increasingly prevalent across the world. A considerable proportion of ticketing websites enable passengers to inquire about the number of remaining tickets (number of seats that are still available for purchase at a given time point) for particular planned airline or railway routes. With the support of application programming interfaces (APIs), one can automatically collect this remaining ticket data (RTD) from websites, making it possible to pool the information for all predefined routes within a predefined time period, thereby producing a unique kind of big data. Clearly, RTD lacks information on individual passengers, hindering any analyses on individual passenger trajectories; on the other hand, this feature has merit in the sense that there is no risk to passenger privacy.

In this paper, we briefly demonstrate how RTD can be used to characterize traveling patterns and the function of transit systems. To this end, we selected a typical nationwide public transit system as our example, the Chinese High-Speed Rail (HSR) system. During the past decade, the Chinese HSR has developed with a conspicuous rate, which has attracted worldwide attention; it has become one of the most important traveling tools for Chinese people. However, the travel patterns associated with the world’s largest HSR system remain poorly understood, due to data restriction. We expect our work will not only facilitate optimizing the Chinese HSR system, but it will also call attention to this unique type of open big data for the study of other analogous transportation systems.

## Materials and methods

China started implementing the large-scale construction of a nationwide HSR system in 2004. By 2016, this system was composed of ~20,000 kilometers of high-speed railways, accounting for 60% of the world’s total length of high-speed railways. According to the *‘Mid- and Long-term Planning on the Railway Network of China*,*’* issued by the Chinese government in 2016, there are ~17,000 kilometers of high-speed railways either currently under construction or in the planning stages. It is projected that, by the year 2020, the HSR network will cover 80% of China’s major cities. The average train speed of Chinese HSR is above 200 km per hour, enabling over 300,000 passengers to travel between the two largest cities of China, Beijing and Shanghai (with a spacing distance of >1300 km), within 4.5 hours. The HSR network strengthens the socioeconomic links between cities, serving as a powerful impetus for China’s rapid development [24].

The HSR ticketing website (www.12306.cn) provides a user-friendly interface that enables passengers to make enquiries on the number of remaining tickets between any two given stations on a Chinese HSR route at any point in time. We developed a specific API using the C# programming language to make such an enquiry automatically. This allowed us to obtain the number of remaining tickets for given scheduled train(s) immediately before departure. We show that at least three types of useful information can be retrieved as follows.

**Fig 1:**
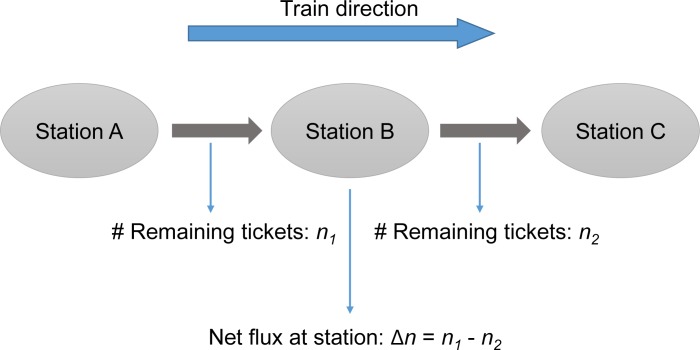
Retrieval of net passenger flux at stations using remaining ticket data. For a given scheduled train passing Station A, B and C sequentially, by enquiring into the numbers of remaining tickets for trips from Station A to B (n_1_), and B to C (n_2_), the net flux at station B can be calculated as their difference (Δn = n_1_—n_2_). Δn>0 represents outflow > inflow at station B, and vice versa.

(1) ‘Raw’ data of remaining tickets. The raw RTD can precisely measure the ‘remaining’ or unused transit capacity of the HSR trains.

(2) Passenger number and occupancy rate. For any given scheduled train, the number of total available seats can be obtained by enquiring about the number of remaining tickets at the very beginning of ticket release (normally 20 days before departure). The passenger number can be estimated as the difference between the total seat number and the number of remaining tickets at the last second before departure. Occupancy rate is calculated as the ratio of the total passenger number to the total seat number.

(3) Passenger flow volume and net flux at individual stations. For intercity trains without intermediate stops, the passenger flow can be retrieved in the same manner as the passenger number described as above. However, this method is not applicable to any situation in which at least one station exists between the origin and destination stations along the route, because the intermediate transits can potentially reduce the share of the remaining tickets. Instead, one can use the RTD to estimate the net ‘flux’ of passengers at a given intermediate station, defined as the volume difference between the inflow and outflow of passengers at this station (Fig 1). Net flux as a functionality indicator reflects the trend if inflow or outflow arises as the dominant behavior at the focal station or railway section.

Clearly it would be difficult to validate the RTD, since the detailed ticketing information is inaccessible at present. We consider the ticketing website trustable, as it is the only official website of Chinese railway authority, but also there is no sign of intentional data manipulation so far.

## Results and discussion

We conducted three sets of analyses as simple case studies to illustrate how the abovementioned three types of RTD can be used to understand traveling patterns on the HSR. The data used for these analyses can be found in S1 File.

### Case 1: Temporal patterns of unused transit capacity

**Fig 2:**
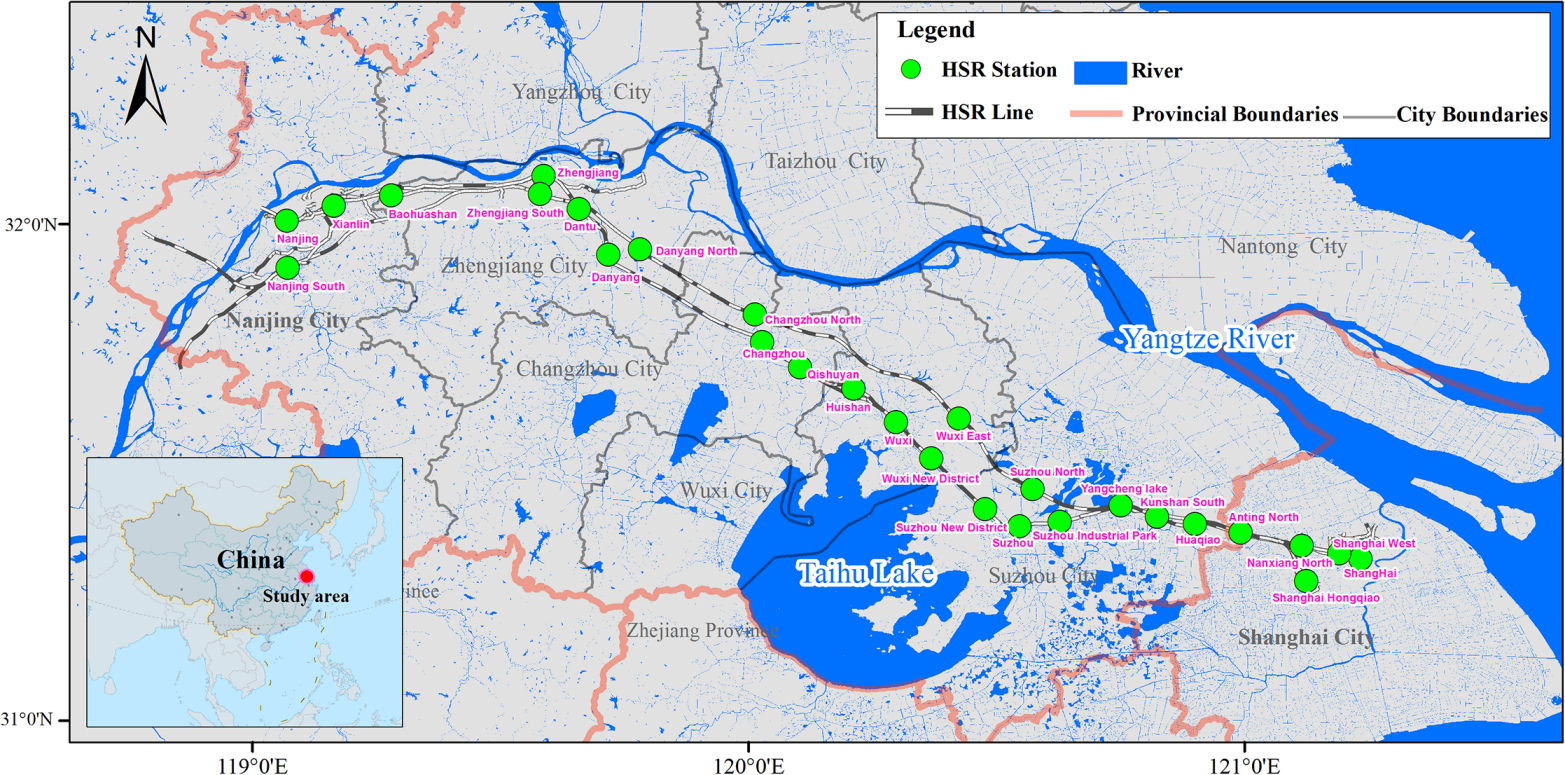
Locations of high-speed railway stations between Nanjing and Shanghai.

Our first case focuses on the Nanjing-Shanghai section in the Chinese HSR network (Fig 2). Nanjing and Shanghai are the two core cities of the Yangtze Delta, which possesses the largest urban agglomeration and the highest population density in China. Currently, the Nanjing-Shanghai section is loaded with the heaviest transit flows across the whole Chinese HSR system, with over 200 trains running on this section every day. Typically, it takes less than 2 hours (3.5 hours at a maximum and 1 hour at a minimum) to travel by HSR between Nanjing and Shanghai (~300 km in distance).

**Fig 3:**
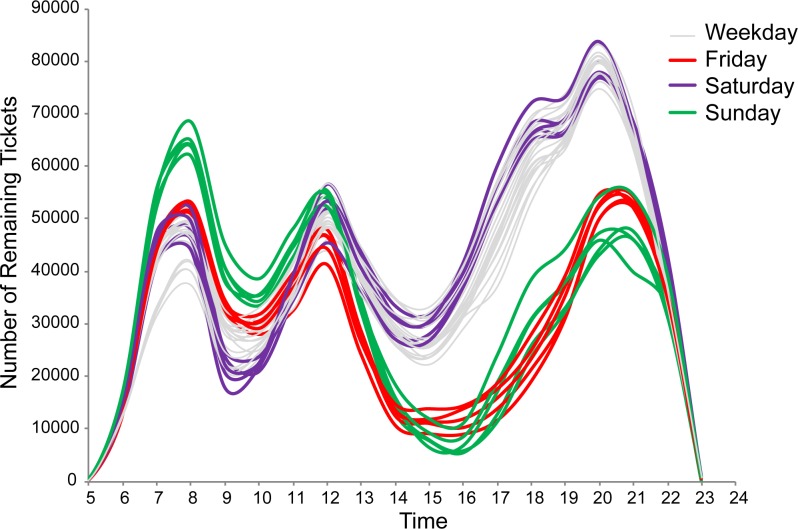
Time series of hourly remaining ticket data for all high-speed trains from Nanjing to Shanghai from November 22—December 26, 2016. Each curve represents a single day.

We collected the hourly RTD for all HSR trains in the direction from Nanjing (origin) to Shanghai (destination) during a time period of 35 days from November 22 to December 26, 2016. The time series of the RTD showed a highly regular intra-day temporal pattern of remaining ticket numbers (Fig 3). One conspicuous feature is the wave-like diurnal cycle, highlighted by two peaks of rush hours around 9–10 a.m. and 3–5 p.m. when very few remaining tickets were available to purchase. This strong temporal pattern can be plausibly explained by the convergence of traveling preference. Travelers in the morning may prefer to finish their travel before noon so that a whole afternoon is available on their schedules, while travelers in the afternoon may prefer to arrive home before dinner to make time for nightlife, rest, etc. Another noteworthy observation is that these rush hours are more significant on Friday and Saturday afternoons (i.e., before the start and end of weekend days), as characterized by a much more intense ticket shortage. This difference reveals a distinctive weekend traveling behavior, which could possibly be induced by long-distance commuters, family visitors, and leisure travelers.

Our simple analysis suggests that, despite high variability in traveling features among individual passengers, their collective behavior presents high temporal regularity as an emergent property. Moreover, this can serve as a starting point in order to address a series of interesting questions in regards to traveling behavior. For example, does the observed pattern still hold for different regions? How do the profiles of remaining tickets respond to important extraneous events? What do these profiles look like if we observe at different temporal (e.g., on a daily, weekly, or monthly basis) and spatial (e.g., for very short- or long-distance travels) scales? Understanding such traveling patterns could have important implications in research areas such as transit system management, public security and the travel economy.

### Case 2: Commuting behavior

Our second case, pertaining to an intercity HSR line between Langfang and Beijing (~50 km in distance), illustrates the value of RTD-derived occupancy rate data. Langfang now serves as a satellite city of Beijing, China’s capital city. Two intercity HSR trains were put into service on November 21, 2014, and were intended to meet the commuting needs of people living in Langfang who worked in Beijing. Every day, a train (HSR #G9002) departs from the Langfang station at 7:25 a.m. and arrives at the Beijing South station at 7:46 a.m.; its counterpart (HSR #G9001) departs from the Beijing South station at 8:41 p.m. and arrives at the Langfang station 9:02 p.m. No other stop exists for these intercity trains.

**Fig 4:**
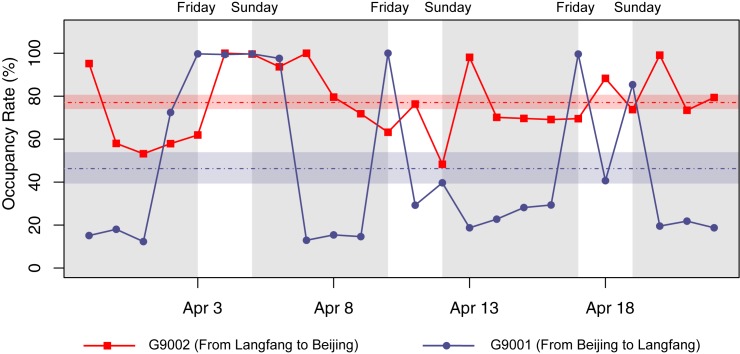
Time series of daily occupancy rates for the two intercity trains G9001 and G9002 between Langfang and Beijing during March 31—April 22, 2015. The horizontal color bands indicate mean occupancy rates with bandwidths indicating standard errors. The gray shaded areas indicate weekdays from Monday to Thursday.

In determining how these trains perform in terms of commuting services as their designed major function, we collected the daily RTD to retrieve occupancy rates during a period of 24 days (from March 31 to April 22, 2015) (Fig 4). On the morning train from Langfang to Beijing, the occupancy rates were usually at a relatively high level, averaging around 80%. In contrast, the evening train in the opposite direction showed a lower average occupancy rate, around 50% with a more fluctuated temporal pattern: Fridays, Saturdays and Sundays typically presented very high occupancy rates >90%. In contrast, the other weekdays maintained low occupancy rates, around 20%. Such differences in the temporal patterns of occupancy rates suggest that there could be a much higher preference for commuters taking the HSR train from home (Langfang) to the work place (Beijing) rather than the other way around. One possible explanation for this imbalanced commuting behavior is that the evening train is simply too late for most commuters to return home during the workweek. In this sense, this profile analysis demonstrates that changing the running times ahead of the current may help improve the evening train’s commuting efficiency.

Similar to the raw RTD in the first case, analyzing the occupancy rate and flow volume at stations along a range of spatial and temporal scales can provide valuable insight into various traveling behaviors. Previous studies based on smart-card data have shown that flow volume data are particularly useful for revealing statistical human mobility patterns [2, 17, 19]. Similar analyses in combination with RTD derived that flow volume data can facilitate a better understanding of the HSR system.

### Case 3: Spatiotemporal patterns of passenger flux

**Fig 5:**
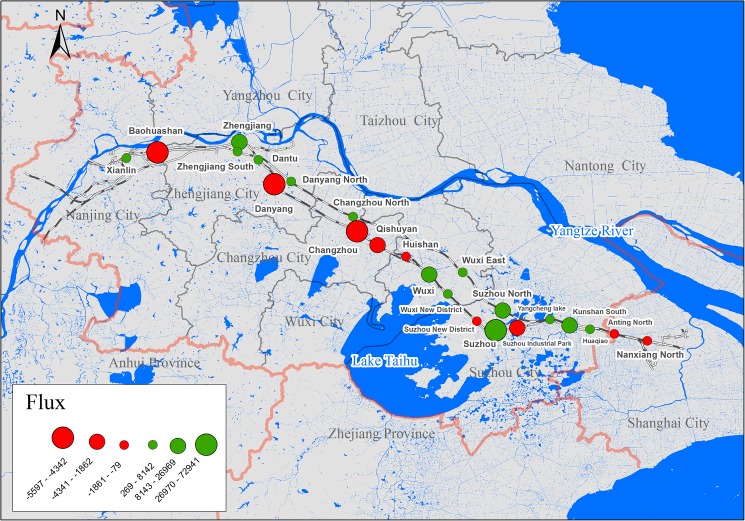
Net passenger flux at high-speed railway stations between Nanjing and Shanghai from August 14 to September 3, 2016. Positive flux value represents outflow > inflow, and vice versa.

Our third case pertains to the net flux at stations in the Nanjing-Shanghai HSR section. We collected the daily RTD for all HSR trains between Nanjing and Shanghai (in both directions) during a period of 21 days, from August 14 to September 3, 2016. The net passenger flux results showed variations of flux between stations (Fig 5). By comparing between the primary and secondary stations [25], our results showed that most primary stations presented relatively high flux (e.g., in the Zhenjiang and Changzhou stations), but this is not always the case, as seen in the Wuxi station, which was comparatively low flux. Such low flux suggests that relatively balanced inflows and outflows cancel each other out, regarding the high flow volume at these stations. Interestingly, some secondary stations (e.g., Baohuashan station) with low flow volume presented considerably high flux, indicating highly imbalanced inflow vs. outflow.

**Fig 6:**
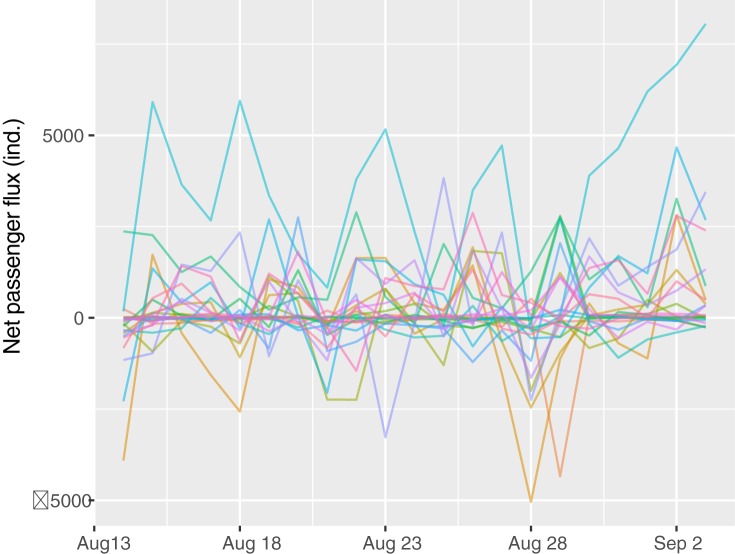
Time series of passenger flux at stations between Nanjing and Shanghai from August 14 to September 3, 2016. Each color curve represents a single station. Positive flux value represents outflow > inflow, and vice versa.

**Fig 7:**
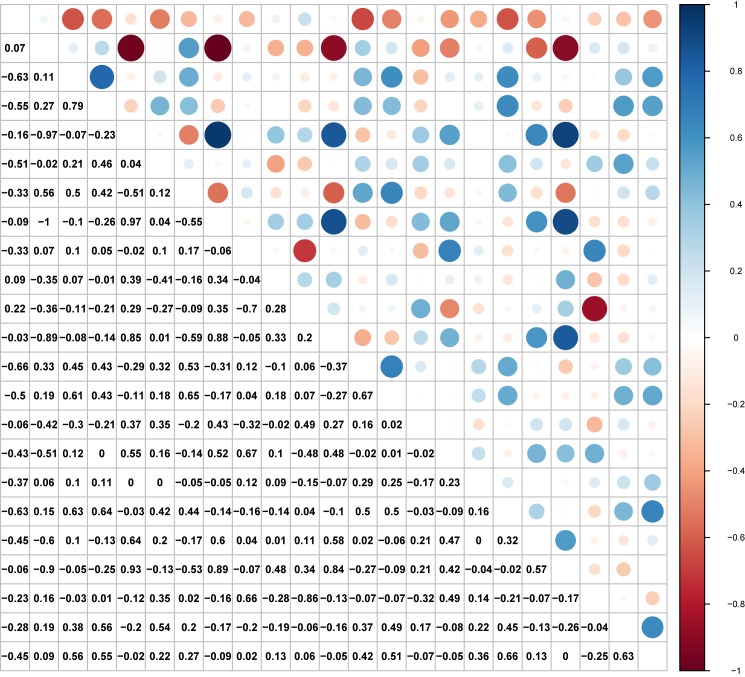
Between-station correlation matrix of passenger flux between Nanjing and Shanghai from August 14 to September 3, 2016. The Pearson’s correlation coefficients are shown.

We observed a fluctuating time series of passenger flux at stations during the 21 days of survey. No visually consistent temporal pattern was detected between the stations (Fig 6); however, we found that a considerable proportion of station pairs manifested strong correlations of net flux with Pearson’s correlation coefficients *r* > 0.6 (Fig 7). Such positive correlations suggested that the dynamics of passenger flux were significantly synchronized between these stations; in other words, these stations may experience similar temporal variations in group size of boarding and alighting passengers. It is no surprise to observe synchronization between the stations within the same cities; however, we also found significantly positive correlations between certain station pairs that are located quite far away from each other, suggesting that these non-adjacent nodes play similarly functioning roles in the HSR network. In contrast, the negative correlations suggest that the corresponding station pairs may function in a complementary fashion, in terms of source vs. sink of passenger flows. Our analyses illustrate that RTD data are potentially valuable for characterizing spatiotemporal patterns of transportation flux across a range of scales and can aid in a better understanding of transit system functionality.

## Concluding remarks

Big data have contributed to deepening our understanding of many human systems, particularly human mobility patterns and the structure and functioning of transportation systems. Thanks to the ‘open big data movement,’ big data are increasingly available in the public domain at the local, regional, and global levels. However, open big data regarding human mobility remain scarce. In this paper we introduced RTD as a useful open big data source. Our case studies are by no means a systematic or comprehensive illustration of the usefulness of RTD. Instead, we intend to use our analyses as exemplifications to call for further exploration into this topic. Through our showcases, we demonstrated a conspicuous wave-like diurnal cycle of passenger flow on the Nanjing-Shanghai HSR section, highlighted by two peaks of ticket shortage in morning and afternoon, respectively. We found marked spatiotemporal heterogeneity in passenger flux across the HSR stations, and strong correlations of net flux suggesting that some stations may play similarly functioning roles in the HSR network. We also revealed imbalanced commuting functions between the morning and evening trains on the intercity HSR line between Langfang and Beijing. Our analyses, albeit simple, demonstrate the power, potential and limitations of RTD for characterizing spatiotemporal traveling patterns. Apart from scientific value, the patterns we found regarding the Chinese HSR can help policy-makers understand and predict travelers’ activity more efficiently, and assess the impact of traffic disruptions or major events, such as sports games, and then take measures to improve transport efficiency and robustness. Our findings could also provide useful implications for optimizing the HSR system in many ways. For example, the observed highly regular temporal pattern of remaining ticket shortage implies that increasing transit capacity in the corresponding time periods could effectively alleviate the short-term transit pressure. Another apparent merit is that RTD can help to assess if particular trains or stations are able to fulfill their designed function (in terms of occupancy rate or passenger flow/flux), so that management authorities can take measures (e.g., rescheduling timetable or re-locating stations) to enhance transit efficiency.

Our analyses, which were based on only a slight fraction of the HSR data, can be extended to larger spatial and temporal scales to obtain a bigger picture of traveling patterns. It would be especially interesting to include all stations to analyze system-wide features of the HSR network. We expect that RTD can generate interesting patterns when applied to a range of existing approaches and analysis frameworks in the studies of human trajectories and social networks, such as jump length distribution, radius of gyration, and measures of complex networks (degree distribution, small-world property, shortest path, etc.) [7, 8, 26–28]; we also expect that the combination of RTD, advanced data mining techniques and other environmental/anthropogenic data [20–23] will contribute to better our understandings on a wide range of social systems. We encourage follow-up studies to address larger spatiotemporal scales and to employ auxiliary data and more in-depth data mining and analysis approaches.

Like any other type of big data, RTD are far from being perfect. First, RTD are generated at the level of individual trains, therefore they cannot be used to retrieve traveling trajectories at the level of individual persons (unlike instances using smart-card data). Second, historical RTD are usually not readily available from transportation websites. Third, flow volume data cannot be precisely retrieved for all individual stations. Fourth, validation of the RTD is still needed to assess if RTD are biased. In addition, there is no user-friendly software for RTD acquisition, researchers therefore have to develop customized computer programs. This technical difficulty may limit further applications of RTD. These merits, limitations and caveats need to be taken into account in future explorations of this unique open big data source.

## Supporting information

S1 File Data.zip. Extracted remaining ticket data used for the analyses.
(ZIP)
